# The Role of 18F-Fluorodeoxyglucose Positron Emission Tomography in the Prognostication, Diagnosis, and Management of Thyroid Carcinoma

**DOI:** 10.1155/2012/198313

**Published:** 2011-10-09

**Authors:** Brian Hung-Hin Lang

**Affiliations:** Division of Endocrine Surgery, Department of Surgery, The University of Hong Kong, Queen Mary Hospital, Pokfulam Road, Hong Kong

## Abstract

18F-fluorodeoxyglucose positron emission tomography (FDG-PET) plays an increasingly important role in the prognostication, diagnosis, and management of thyroid carcinoma. For patients diagnosed with primary or persistent/recurrent thyroid carcinoma, a finding of FDG-PET positivity implies a more aggressive tumor biology and a distinct mutational profile, both of which carry prognostic significance. Therefore, FDG-PET positivity may be a useful potential risk factor for preoperative risk stratification in primary thyroid carcinoma. This information may help in the planning of subsequent treatment strategy such as the extent of thyroidectomy, prophylactic central neck dissection, and radioiodine ablation. FDG-PET scan has also been found to be a useful adjunct in characterizing indeterminate thyroid nodules on fine needle aspiration cytology. However, larger-sized prospective studies are required to validate this finding. FDG-PET or FDG-PET/CT scan has become the imaging of choice in patients with a negative whole-body radioiodine scan, but with an abnormally raised thyroglobulin level after total thyroidectomy and radioiodine ablation.

## 1. Introduction

18F-fluoro-2-deoxy-D-glucose-positron emission tomography (FDG-PET) scan is increasingly being used not only in oncology but also in some nononcological specialties, such as neurology, cardiology, and infectious diseases [1]. The fundamental principle of FDG-PET scan is that the nuclide, 18F-FDG has two parts, namely, the vector part (i.e., 2-deoxy-D-glucose) and the positron emitting nuclide part (i.e., 18F), and when it gets preferentially taken up by rapidly dividing cells (i.e., malignant cells), it gets “trapped” within the cells and emits positron radiation which is then detected by the scintigraphy [2]. This is called metabolic trapping and this forms the basis for FDG-PET scanning. In oncology, FDG-PET has many important clinical applications including initial cancer staging as well as monitoring tumor response to anticancer therapy in lung, colon, lymphoma, melanoma, esophageal cancer, and head and neck and breast cancer [3]. In neurology and cardiology, FDG-PET is a useful tool for localization of epileptogenic zones inside the brain and is a “gold standard” tool for the detection of myocardial viability, respectively [4, 5].

Since the first observation of FDG uptake in metastatic thyroid cancer over 20 years ago [6], there have been growing interests in evaluating the role of FDG-PET scanning in the management of thyroid neoplasms [7]. Also with an increased number of FDG-PET scans now being performed, an increasing number of incidental thyroid lesions (or the so-called thyroid incidentalomas) have been found and this itself poses a diagnostic challenge to clinicians. Given that FDG-PET imaging could provide potentially relevant information on tumor biology and the scan results may enable to prognostically stratify thyroid carcinoma patients, the author believes that it would be both timely and important to examine the prognostic significance of FDG-PET positivity in thyroid carcinoma and the role of FDG-PET in diagnosis and management of thyroid carcinoma by a review of the current literature.

## 2. Prognostic Significance of FDG-PET Positivity or FDG Avidity in Thyroid Carcinoma

It has been well recognized that thyroid carcinoma metastases which are shown up on FDG-PET scan do not take up radioactive iodine (RAI) [7]. In fact, it was noted that there was a reciprocal or “flip-flop” relationship between RAI and FDG uptake [6]. Numerous reports have confirmed this important relationship [8]. At the same time, it was noted that those metastases which did not take up RAI were generally less differentiated on histology and behaved clinically more aggressively. Therefore, FDG-PET positivity in thyroid carcinoma could imply clinically more aggressive tumors and poorer overall prognosis. However, before discussing the implications of FDG positivity in thyroid carcinoma, it should be realized that there are fundamental differences in the management of FDG-PET positivity between primary and persistent/recurrent thyroid carcinoma. As a result, the following discussion was subdivided into primary and persistent/recurrent thyroid carcinoma. 

## 3. Prognostic Significance of FDG-PET Positivity in Primary Thyroid Carcinoma

**Figure 1:**
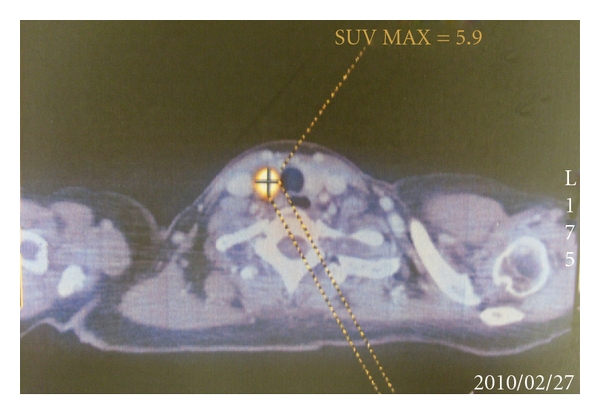
A 70-year-old lady underwent 18F-FDG PET for staging of a rectal carcinoma. A focal right thyroid hypermetabolic uptake (with a SUVmax of 5.9) was detected. This was later confirmed as papillary thyroid carcinoma (PTC) on cytology. A total thyroidectomy was performed revealing a 1.3 cm tall cell variant of PTC with central compartment lymph nodes metastases (pT1N1a).

Unlike persistent/recurrent carcinoma, the number of studies examining the relationship between FDG-PET positivity and tumor behavior or prognosis for primary thyroid carcinoma remained relatively few. As the role of FDG-PET in preoperative thyroid carcinoma staging remains undefined, the majority of these primary thyroid carcinomas exhibiting FDG-PET positivity are incidentally found cancers or incidentalomas (see Figure 1). Reasons for the lack of enthusiasm for FDG-PET as a staging tool include that FDG-PET scan remains a relatively expensive preoperative imaging modality when compared to ultrasound (USG) which is the recommended imaging modality before surgery, and relative to the other imaging modalities, FDG-PET does not seem to provide any additional staging information (such as the status of the cervical lymph nodes) to an extent of altering the surgical management [9]. As a result, FDG-PET scan has not been routinely advocated as a preoperative staging tool, although there might be a role in selected, more aggressive pathologies, such as Hurthle cell or anaplastic thyroid carcinoma [10–12]. Nevertheless, one of the first studies examining the relationship between tumor behavior and FDG-PET positivity in primary thyroid carcinoma was reported by Jeong et al. [13]. In this study, they reported 44 consecutive patients with papillary thyroid microcarcinoma (PTMC) confirmed by USG and fine needle aspiration cytology (FNAC) who subsequently underwent FDG-PET scans before surgery. The clinicopathological characteristics of these 44 PTMC were correlated with the activity of the FDG-PET scan or FDG standardized uptakes values (SUVs). Although there was a strong correlation between SUVs and extrathyroidal extension of PTMC in the univariate analysis, the study did not find any association between the degree of SUV and extrathyroidal extension or other aggressive tumor features in the multivariate analysis [13]. Only age > 45 and tumor site turned out to be the two significant factors for determining extrathyroidal extension in primary PTMC [13]. SUVs were correlated with tumor size, but this was not unexpected because there is the partial volume effect between small- and large-sized lesions. Therefore, based on this initial study, FDG-PET positivity was not associated with more aggressive tumor behavior or worse tumor characteristics [13]. However, a more recent study found that for PTMCs which were FDG nonavid, they were not only significantly smaller sized tumors but also less frequent perithyroidal tumor invasion and lymphovascular invasion when compared to the PTMCs which were FDG avid [14]. This implies that FDG-PET positivity might be associated with a more aggressive tumor behavior in primary thyroid carcinoma [14]. However, since tumor size is an important factor for FDG-PET positivity as it relates to the partial volume effect, it would be more appropriate to adjust for tumor size in these clinicopathological studies. Yun et al. reported their retrospective study involving 87 patients with a unifocal PTMC who underwent preoperative FDG-PET before total thyroidectomy and central neck dissection [15]. They defined positive FDG uptake in PTMCs as a discernible focal FDG uptake whereas negative FDG as no discernible FDG uptake. All scans were assessed by two experienced nuclear medicine specialists blinded for patients' clinical and pathological variables. In their multivariate analyses, among other factors such as gender, age, and tumor size, FDG-PET positivity was the only significant factor which strongly correlated with extrathyroidal extension (OR = 5.95; 95% CI: 2.13–16.6) and central lymph node metastases in primary PTMC [15]. This result indicates that visual FDG-PET positivity in PTMCs is a potential risk factor which could be useful in preoperative risk stratification. One of the potential clinical applications would be to use the preoperative finding of FDG-PET positivity to select patients for more extensive thyroid resection (i.e., hemithyroidectomy versus total thyroidectomy in PTMC) or for prophylactic central neck dissection at the time of total thyroidectomy as it is currently only indicated for high-risk tumors [10]. Our previous studies examining the behavior of FDG-avid primary thyroid carcinoma (mostly incidental thyroid carcinoma) also suggested that these tumors are not only larger in size, more likely to be clinically significant (42.9% versus 2.9%, *P* = 0.001) but also more aggressive in terms of having higher frequency of tumor bilaterality (45% versus 0%, *P* = 0.040) when compared to non-FDG-avid tumors [16, 17]. In fact, we advocated a total thyroidectomy even for FDG-avid thyroid carcinoma <1 cm in diameter because of the high incidence of tumor bilaterality [16]. Therefore, our data would support the fact that FDG-PET positivity implies more aggressive tumor biology and poorer prognosis in primary thyroid carcinoma.

## 4. Prognostic Significance of FDG-PET Positivity in Persistent or Recurrent Thyroid Carcinoma

Numerous studies have found that metastases from thyroid carcinoma which do not concentrate RAI but take up FDG are clinically more aggressive and have poorer tumor differentiation [7]. Esteva et al. studied 50 differentiated thyroid carcinoma (DTC) patients with elevated thyroglobulin (Tg) and negative whole-body scan (WBS) after total thyroidectomy and RAI ablation. All patients underwent a FDG-PET scan one week after the WBS [18]. The authors correlated the postoperative FDG-PET finding and clinicopathological variables of the primary tumor and found that FDG-PET was positive in 32/39 patients with confirmed persistent or recurrent thyroid carcinoma. When compared to the FDG-PET-negative group, the FDG-PET-positive group had significantly larger primary tumor size (2.82 cm versus 1,72 cm, *P* < 0.05) and these primary tumors were more likely to have capsular invasion (62.5% versus 16.7%, *P* < 0.05) suggesting that more advanced primary tumors were more likely to have FDG-PET-positive recurrences [18]. Rivera et al. studied the histology of the metastases from 70 patients with RAI refractory but FDG-PET-positive recurrences [19]. Of these 70 patients, 33 (47.1%) had poorly DTC, 16 (22.9%) had well DTC, 6 (8.6%) had Hurthle cell carcinoma, and 1 (1.4%) had anaplastic carcinoma. Based on these findings, the majority of RAI refractory and FDG-PET positive metastases are of a histological aggressive subtype. Interestingly, when the histology of the primary tumor and its subsequent metastases was matched, in most instances, there was a gradual transformation from well-differentiated histology to less-differentiated histology over time, and this might be the reason why even if the primary tumor might not be FDG avid initially, its metastases become FDG avid over time [19]. This phenomenon was somewhat supported by a recent study which found that *BRAF* mutations represented early events in thyroid carcinogenesis, whereas mutations of *PIK3CA* and *AKT1* were latter events not found in the primary cancers, but in metastases or recurrent cancers [20].

Apart from the direct relationship between FDG-PET positivity and poorer histological differentiation in persistent or recurrent thyroid carcinoma, it was observed that patients with RAI-refractory but FDG-avid metastases were significantly more likely to die from thyroid carcinoma than those with RAI-refractory and non-FDG-avid metastases. This remained true when tumors were matched for *TNM* stages. Using a Cox proportional hazard model, Robbins et al. found that age (RR = 1.33; 95% CI: 1.08–1.52), FDG status (RR = 7.69; 95% CI: 2.17–24.4), and number of FDG lesions (RR = 1.1; 95% CI: 1.08–1.15) significantly correlated with cancer-specific survivals [21]. Therefore, one could conclude that FDG-PET positivity in persistent/recurrent thyroid carcinoma and the number of FDG lesions are highly prognostic for survival [21, 22].

## 5. Molecular Basis of FDG-PET Positivity in Thyroid Carcinoma

Given the fact that mutations in significant oncogenes such as *BRAF*, *TP53* (all of which are able to activate the mitogen-activated protein kinase pathway) are often present in aggressive histological subtypes, it is reasonable to assume that both primary tumors and their metastases which are FDG-PET positive would have a unique mutational profile. Ricarte-Filho et al. found that 100% of FDG-PET positive and RAI refractory tumors carried *BRAF *mutations, whereas in general only 45% of DTC would carry such mutations [20]. This led to the postulation that perhaps there is a causal relationship between *BRAF* mutations and FDG-PET positivity. For the lack of RAI uptake in FDG-avid tumors, it was shown for the first time that in a DTC cell line, the conditional activation of BRAF^V600E^ tended to downregulate the expression of the sodium iodide symporter (NIS) which is an important ion pump for the transport of iodine across basolateral membrane [23]. This finding was later confirmed in several studies showing that BRAF^V600E^ was associated with reduced *NIS* and *NIS* mRNA expression [24]. One of the proposed mechanisms through which *BRAF* represses *NIS* is by the induction of robust transforming growth factor (TGF) *β* secretion and subsequent activation of TGF*β*/Smad signaling [25]. However, these findings would only explain why these FDG-avid tumors do not take up RAI. To establish the possible association between *BRAF* mutations and FDG-PET positivity, Durante et al. examined the expression of several key markers of thyrocyte differentiation including *NIS*, Tg, thyroperoxidase, TSH receptor, transcription factor *PAX8*, and glucose transporter type I (GLUT1) in 56 papillary thyroid carcinoma (PTC) with *BRAF* mutations (i.e., *BRAF*-positive), 37 with no *BRAF* mutations (i.e., *BRAF*-negative), and 8 normal thyroid tissue [26]. Relative to normal thyroid tissue, all markers including GLUT1 in PTCs were reduced, but more importantly, there were additional increases in GLUT1 mRNA when only the *BRAF*-positive tumors were selected [26]. It is now believed that among the four GLUT isoforms, GLUT1 is the most prevalent isoform responsible for FDG-PET positivity in less-differentiated thyroid carcinomas [27].

## 6. Role of FDG-PET in the Diagnosis of Thyroid Carcinoma

Since histologically benign and malignant thyroid lesions do exhibit some differences in SUV, FDG-PET may have an important role in the characterization of thyroid nodules and more specifically in the diagnosis of thyroid carcinoma. However, currently the most cost-effective and accurate way of making the diagnosis of thyroid carcinoma relies on clinical examination, the use of USG, and USG-guided FNAC. Therefore, FDG-PET has not been widely accepted as a diagnostic tool in thyroid carcinoma, but this may change with time as technology improves [36]. One aspect of diagnosis in which FDG-PET scan has shown some promises is in patients with an indeterminate thyroid lesion on FNAC. By definition, an indeterminate FNAC usually includes follicular lesions, Hurthle cell (oncocytic) lesions, atypical cytology, abnormal cytology, or suspicious cytology. This group remains a diagnostic dilemma to clinicians because approximately 20–30% of these will be malignant, whereas the rest will be benign by pathological examination [37, 38]. However, nearly all patients with indeterminate FNAC would be required to undergo thyroid lobectomy to establish the diagnosis. In other words, surgical resection will prove unnecessary in over 60–70% of cases. At present, there is no alternative algorithm for a more conservative management for patients with indeterminate FNAC. Convectional USG, computed tomography (CT), and magnetic resonance imaging (MRI) have been previously shown to be of some value, but FDG-PET is yet to be assessed in a large prospective study [34].  Table 1 shows a comparison of series examining the utility of FDG-PET in detecting malignancy in thyroid nodule with indeterminate FNAC. Study size ranged from 15 to 51 patients, but the inclusion criteria varied between studies. One of the larger series was reported by de Geus-Oei et al. [30]. There were 44 patients with inconclusive FNAC (i.e., follicular neoplasm, Hurthle cell neoplasm, atypical cells, or inadequate) and of these, 6 were malignant and the other 38 were benign. In their experience, FDG-PET did not lead any false negatives implying a negative predictive value (NPV) of 100%. All carcinomas demonstrated FDG uptake, but 13 of 38 benign nodules also had FDG uptake. They showed that using routine FDG-PET in their patient cohort could potentially reduce the number of unnecessary lobectomies by 66% (95% CI: 49–80%) if surgery was not advised for those with no uptake in the thyroid gland on FDG-PET imaging [30]. Importantly, no malignancies were missed (i.e., no false negatives), but still there were 13/38 (34.2%) who underwent unnecessary lobectomies (i.e., false positives). It was interesting to note in their series that the mean SUV was similar between benign and malignant lesions and one of the PTCs actually had SUV as low as 0.9. To improve the false-positive rate, some studies proposed using focal uptake SUV > 2.0 as the criterion for FDG-PET positivity [28, 32]. However, for whatever cut-off in SUV, there is always going to be a trade-off between false positives and false negatives. Smith et al. evaluated the association between SUV uptake over time and malignancy in follicular neoplasm by performing serial FDG-PET scans over a 2-hour period and measuring the area under the SUV curve (AUC) [33]. They demonstrated no significant difference in AUC, but found a difference in the dynamics of SUV change over time between benign and malignant lesions [32]. Due to the significant overlap in AUC, they concluded that FDG-PET was not able to predict malignancy in a follicular neoplasm [32]. However, since most of these reported series were retrospective in design and the patient selection was not formally standardized, there is a need for a large, well-designed prospective study. Recently, Traugott et al. reported the results of 51 patients with indeterminate FNAC as an interim analysis of their prospective trial which began in 2004 [34]. Their data suggested that FDG-PET was an accurate diagnostic modality for identifying malignancy in thyroid nodules at least 1 cm in diameter and with indeterminate FNAC, with 100% sensitivity and NPV. They concluded that FDG-PET was of value in excluding malignancy in thyroid nodules with indeterminate FNAC. To overcome the spatial resolution limitation of the FDG-PET, their study only included solitary or dominant nodule that measured ≥1 cm on USG. To achieve adequate power, they aimed to recruit 125 patients and results would be available within the next few years. A recent systematic review and meta-analysis evaluated the role of FDG-PET in patients with indeterminate thyroid FNAC [35]. In this meta-analysis, they analyzed over 200 patients and found that the pooled sensitivity, specificity, PPV, and NPV were 95%, 39%, 96%, and 60%, respectively. The authors concluded that a negative FDG-PET scan in patients who had thyroid nodules > 15 mm with indeterminate FNAB results excluded thyroid cancer [35]. Conversely, a positive FDG-PET result did not identify cancer because approximately 50% of these patients had benign nodules [35]. They concluded that the incorporation of FDG-PET into the initial workup of such patients before surgery deserves further investigation [35]. However, there are some concerns using FDG-PET in this group of patients. Firstly, follicular thyroid carcinoma (FTC), which accounts for 10–20% of malignancy in the indeterminate group, is known to be associated with a lower SUV than other histological types of thyroid carcinoma [39]. It is also known that Hurthle cell adenoma, a benign lesion, tends to have very high SUVs [28]. Both entities could possibly lessen the usefulness of FDG-PET in discriminating benign from malignant lesions in this indeterminate group. Furthermore, a confirmatory diagnosis in this group of FNAC is often difficult to make by experienced pathologists. It is known that considerable interobserver and intraobserver variability in the histopathological diagnosis of thyroid follicular lesions has been reported [40, 41]. In one recent study, among 15 cases of suspected follicular variant of PTC (FVPTC), only 2 cases (13.3%) had unanimous agreement among 6 expert pathologists [41]. Given these concerns, the role of FDG-PET in thyroid nodules with indeterminate FNAC remains uncertain, but the interim results in one of the possibly largest prospective trials on FDG-PET in cytologically indeterminate nodules looked promising [34]. Hopefully, this prospective trial which is due to complete in the next few years will provide us with more information.

## 7. Role of FDG-PET in the Management of Thyroid Carcinoma

**Figure 2:**
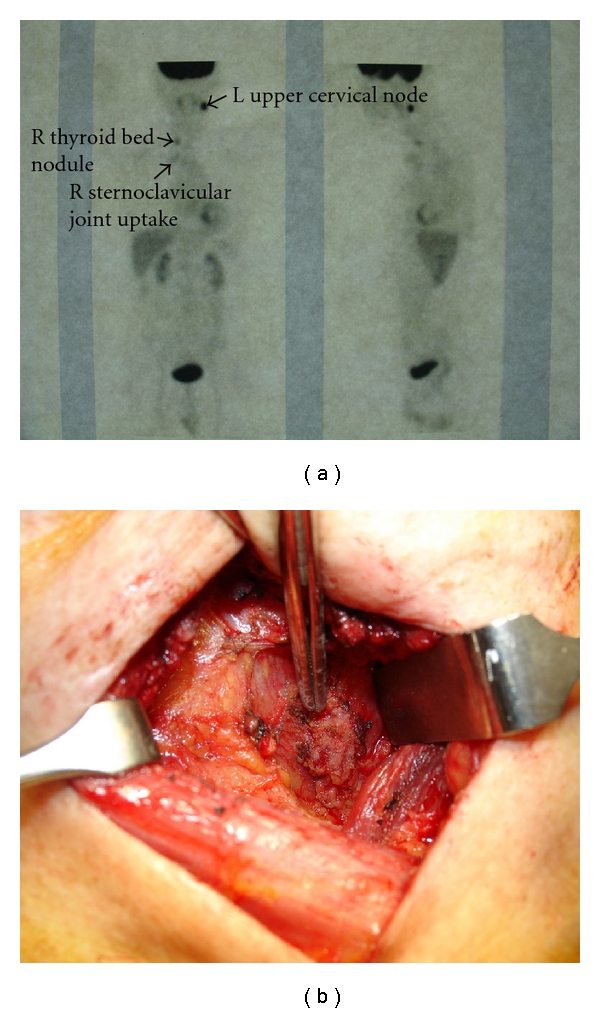
18F-FDG-PET of a 73-year-old man with an 8-year history of papillary thyroid carcinoma. The serum thyroglobulin was elevated at 66 ug/L (normal < 55 ug/L), but both diagnostic whole-body ^131^I scan and ultrasound were negative for recurrence. (a) FDG-PET revealed hypermetabolic uptakes in left cervical lymph node, right thyroid bed, and right sternoclavicular joint area. (b) An operative picture confirming the presence of tumor recurrence at the right thyroid bed (as pointed by the tip of the forceps).

Routine RAI diagnostic scanning or WBS for thyroid carcinoma surveillance is becoming less frequently used because of its relatively low sensitivity and has been supplanted by serum Tg level and neck USG. Nowadays, one of the commonest clinical scenarios would be for a patient with negative or normal USG but raised unstimulated Tg (i.e., >10 ng/mL or 10 ug/L) [10]. FDG-PET is recommended in patients with suspected recurrence or metastases in the setting of raised Tg levels and scan-negative metastases (see Figures 2(a) and 2(b)) [10]. Earlier studies found that FDG-PET was a useful diagnostic tool in the followup of postsurgical patients with DTC, negative WBS, and abnormal Tg levels [42]. FDG-PET was able to detect metastatic disease over 90% of cases and more importantly, it was able to change the surgical tactic in a 20–30% of cases [42]. Similarly, Wang et al. reported their experience of 37 DTC patients with negative WBS and elevated Tg, found that FDG-PET was able to locate occult disease in 71%, and reported a positive predictive value (PPV) of 92%. More importantly, FDG-PET was able to change the clinical management in over 50% of patients [43]. In the presence of low Tg levels, FDG-PET had the NPV of 93% [43]. Esteva et al. reported the FDG-PET findings in 50 patients with elevated Tg and negative WBS and found that FDG-PET was positive in 32/39 (82.1%) patients with confirmed recurrence and negative in 7/11 (63.6%) with no confirmed recurrence. The sensitivity and specificity were 82% and 64%, respectively [18]. Tumor size and capsular tumor invasion were factors significantly associated with a positive FDG-PET study [18]. They also concluded that FDG-PET was an extremely useful imaging tool in patients with negative WBS and raised Tg [18]. However, the added value of FDG-PET/CT over good-quality conventional imaging modalities such as USG, CT, MRI, and diagnostic WBS in locating recurrent or persistent DTC has recently been questioned mainly of the extra cost with FDG-PET/CT and the associated radiation. Lal et al. recently evaluated the added value of FDG-PET/CT over conventional imaging studies in 20 DTC patients with elevated Tg and negative diagnostic WBS [44]. They found FDG-PET/CT provided additional information in only 2/20 (10.0%) patients, both of whom required no additional intervention, but underestimated the extent of disease in 3/30 (15.0%) patients and led to unnecessary interventions (including surgery, RAI, and antibiotics) in 3/30 (15.0%) additional patients. They concluded that FDG-PET/CT has a good sensitivity in detecting recurrent or persistent DTC, but the added value over good-quality conventional imaging is very limited [44]. Furthermore, it may lead to unnecessary interventions [44]. Perhaps, future large prospective studies are required to resolve some of the controversies.

**Figure 3:**
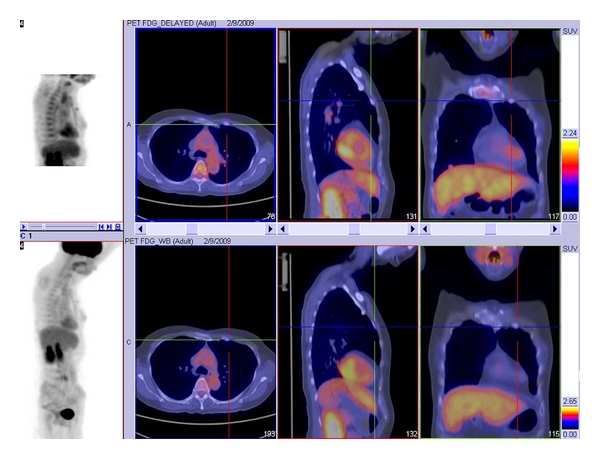
18F-FDG-PET/CT of a 57-year-old woman with a 10-year history of widely invasive follicular thyroid carcinoma. Serum thyroglobulin was elevated to 121 ug/L (normal < 55 ug/L), but both diagnostic whole-body ^131^I scan and ultrasound were negative. The FDG-PET/CT revealed hypermetabolic FDG uptakes in the rib cage.

Some studies evaluated the relationship between TSH level and FDG uptake intensity [45–47]. To date, the evidence seems to suggest that a higher SUVmax could be obtained in TSH-stimulated condition by recombinant human TSH (or rhTSH) stimulation, and as a result of high level of TSH, greater number of FDG-avid metastases could be detected on scanning [45–47]. Some authors evaluated the diagnostic accuracy of integrated FDG-PET/CT as it was believed that fusion of the two modalities may further enhance the sensitivity, specificity, and tumor localization (see Figure 3). For other types of head and neck tumors, combined FDG-PET/CT has been shown to have improved diagnostic accuracy than FDG-PET or CT alone [48]. Razfar et al. evaluated the diagnostic accuracy of integrity of FDG-PET/CT in detecting recurrent/persistent DTC. They reported the sensitivity, specificity, PPV, and NPV to be 80.7%, 88.9%, 94.7%, and 65.3%, respectively. From their analysis, they demonstrated there was an alteration in the treatment strategy in 28.2% as a result of adding the FDG-PET/CT information, and 21% required additional surgery [49]. Therefore, it would seem that FDG-PET/CT scan might be superior to FDG-PET as the imaging of choice in patients with a negative whole-body radioiodine scan and an abnormally raised thyroglobulin level after total thyroidectomy and radioiodine ablation.

## 8. Conclusion

In patients with either primary or persistent/recurrent thyroid carcinoma, the finding of FDG-PET positivity or FDG-avidity usually implies poorer tumor differentiation, more aggressive tumor biology, and worse prognostic outcomes. These observations are supported by the unique mutational profile of FDG-avid tumors or metastases, namely, increased frequency of *BRAF* mutations leading to decreased *NIS* and increased *GLUT1*. FDG-PET positivity may be a useful potential risk factor for preoperative risk stratification in primary thyroid carcinoma and this information may help in the planning of subsequent treatment strategy such as the extent of thyroidectomy, prophylactic central neck dissection, and RAI ablation. FDG-PET scan has the potential in characterizing indeterminate thyroid nodules on FNAC. However, larger-sized prospective studies are required to validate this finding. FDG-PET or FDG-PET/CT scan has become the imaging of choice in patients with a negative WBS, but with an abnormally raised Tg level after total thyroidectomy and RAI ablation.

## Figures and Tables

**Table 1:** A comparison of studies examining the utility of FDG-PET in detecting malignancy in cytologically indeterminate thyroid nodules.

First author (year)	No. of patients	No. of carcinoma (%)	Definition of FDG-PET positivity	Sensitivity (%)	Specificity (%)
Kresnik (2003) [28]	24	9 (37.5)	Focal uptake with SUV > 2	100	100
Mitchell (2005) [29]	24	11 (45.8)	Focal uptake	60	91
de Geus-Oei (2006) [30]	44	6 (13.6)	Focal uptake	100	66
Sebastianes (2007) [31]	42	11 (26.2)	Focal uptake	100	39
Hales (2008) [32]	15	7 (46.7)	Focal uptake with SUV > 2	57	50
Smith (2008) [33]	23	5 (21.7)	Area under SUV curve > 175.5	100	44
Traugott (2010) [34]	51*	8 (15.6)	Focal uptake	100	59
Vriens (2011) [35]	225^#^	58 (25.8)	Varied	94.8	47.9

FDG-PET: fluorodeoxyglucose positron emission tomography; SUV: standardized uptake value.

*An ongoing prospective trial; ^#^a meta-analysis.
